# Editorial for the Special Issue “Interactions Between Probiotics and Host”

**DOI:** 10.3390/microorganisms14081727

**Published:** 2026-08-06

**Authors:** Sávio Sandes

**Affiliations:** Departamento de Ciências de Alimentos e Nutrição, Faculdade de Engenharia de Alimentos, Universidade Estadual de Campinas (UNICAMP), Rua Monteiro Lobato, No. 80, Bairro Cidade Universitária, Campinas 13083-862, SP, Brazil; savio.cicco@gmail.com

The Special Issue “Interactions Between Probiotics and Host” brings into sharp focus a field that has rapidly evolved from descriptive microbiology to a mechanistically grounded and translationally relevant discipline. The seven contributions gathered here collectively underscore a central theme: the intricate and specific interplay between microbes, their bioactive products, and host physiology [1,2,3,4,5,6,7].

A notable thread running through this issue is the shift from microorganisms toward defined bioactive components and metabolites [1,2]. The study introducing an innovative postbiotic derived from sequential fermentation of *Lacticaseibacillus* strains exemplifies how biotechnological processes can enhance functional outcomes [1]. By demonstrating synergistic effects on epithelial barrier integrity and innate immune responses, this work highlights the potential of engineered postbiotics as next-generation interventions. Complementing this, the comprehensive review of postbiotic proteins such as p40, p75, and HM0539 consolidates growing evidence that discrete microbial molecules can recapitulate—and in some contexts refine—the beneficial effects traditionally attributed to live probiotics [2].

Mechanistic insight is further deepened by investigations into host–microbe signaling pathways. The elucidation of TLR9-mediated activation of plasmacytoid dendritic cells by bacterial DNA provides a compelling example of how specific microbial components engage innate immune sensors to shape antiviral defenses [3]. Such findings move the field beyond associative observations, offering precise molecular targets that could inform the design of functional foods and therapeutics.

At the organismal level, preclinical and clinical studies included in this issue reinforce the systemic implications of probiotic interventions [4,5,6,7]. The protective effects of fermented milk containing *Lacticaseibacillus rhamnosus* D1 against *Salmonella* infection in mice illustrate how microbiota modulation, immune regulation, and barrier function converge to influence disease outcomes [4]. Similarly, the pilot clinical trial on oral health extends the relevance of probiotics beyond the gut, suggesting that modulation of local microbiota and inflammatory markers can yield tangible benefits in conditions such as oral malodor [5].

Broadening the scope of probiotic research, the inclusion of yeast-based systems challenges the bacterial-centric paradigm that has long dominated the field. Advances in the production and formulation of yeast probiotics, alongside their diverse mechanisms of action, point to an expanded repertoire of microbial candidates with applications that span gastrointestinal, dermatological, and respiratory health [6].

Finally, the exploration of the gut–brain axis in neurosurgical contexts reflects an emerging frontier where microbiome research intersects with complex clinical scenarios [7]. While current evidence suggests promising roles for probiotics in modulating inflammation, infection, and recovery, the variability in outcomes underscores the necessity for rigorously designed, strain-specific clinical trials.

Taken together, the contributions in this Special Issue highlight both the progress achieved and the challenges that remain. A recurring message is the importance of specificity—of strains, molecules, host contexts, and clinical indications. Future research will need to integrate mechanistic precision with clinical rigor, embracing interdisciplinary approaches that bridge microbiology, immunology, bioengineering, and medicine.

As the field advances, the concept of “interactions” between probiotics and the host should be viewed not as a simple binary relationship, but as a dynamic network of signals and responses. Deciphering this network holds the key to unlocking targeted, effective, and safe microbiome-based interventions. This Special Issue represents a meaningful step in this direction, offering insights that will inform both foundational science and translational innovation.
